# The Diagnostic Challenge of a Dyshidrosiform Bullous Pemphigoid: A Palmar Puzzle

**DOI:** 10.7759/cureus.66470

**Published:** 2024-08-08

**Authors:** Kritin K Verma, Ethan Matthew, Fatma Z Deligonul, Kristina Blegen, Michelle Tarbox

**Affiliations:** 1 Medicine, Texas Tech University Health Sciences Center, Lubbock, USA; 2 Dermatology, Texas Tech University Health Sciences Center, Lubbock, USA; 3 Osteopathic Medicine, University of the Incarnate Word, San Antonio, USA

**Keywords:** dyshidrosiform bullous pemphigoid, autoimmune blistering disease, elderly patient, hemorrhagic blisters, direct immunofluorescence, corticosteroid treatment, alzheimer's disease, palmoplantar lesions, immunosuppressive therapy, case report

## Abstract

We present a unique case of an 89-year-old male with Alzheimer's disease who developed hemorrhagic blisters on his palms, which ruptured with time and were followed by pruritic erythematous lesions across his chest, upper back, lower abdomen, and thighs. The patient was diagnosed with dyshidrosiform bullous pemphigoid (DBP), an uncommon variant of the autoimmune condition bullous pemphigoid characterized by cutaneous and mucosal blistering, which commonly appears as vesiculobullous eruptions in the palmoplantar areas and may spread to other parts of the body. Less than 100 cases of DBP have been documented in the medical literature. Since DBP is difficult to identify and treat due to its clinical appearance similar to pompholyx, we reviewed the treatment of DBP and included clinical images and direct immunofluorescence (DIF) staining technique images to better establish the diagnosis.

## Introduction

Dyshidrosiform bullous pemphigoid (DBP) is a rare variant of bullous pemphigoid, an autoimmune blistering disease that predominantly causes cutaneous and mucosal blistering [1]. This particular variant of bullous pemphigoid is characterized by pruritic blisters on the palms and soles of elderly individuals, typically between the ages of 60 and 90 [1]. These blisters may be hemorrhagic or purpuric and are usually confined to the palms and soles; however, lesion presentation may extend to other parts of the body [2,3]. The appearance of the blisters can range from edematous and erythematous to bullous [1]. Additionally, they may also be accompanied by bleeding or smaller dyshidrosiform vesicles [4].

The pathophysiology of DBP involves circulating and tissue-bound autoantibodies directed against bullous pemphigoid antigen 1 (BP180), bullous pemphigoid antigen 2 (BP230), or both [1]. These antigens are hemidesmosome structural elements that are necessary to preserve basal keratinocyte attachment to the dermal extracellular matrix [1,2]. Subepidermal blisters, which are indicative of the condition, occur when autoantibodies attach to these antigens and set off an inflammatory cascade [1,2]. Accurate diagnosis of DBP can be difficult since its clinical presentation frequently resembles that of dyshidrotic eczema [2].

Since it was first documented in the medical literature in 1979, there have been slightly fewer than 100 reported cases of DBP [1,2]. The presentation of DBP can often mimic dyshidrotic eczema, making an accurate diagnosis difficult; therefore, we want to demonstrate another case of this complex condition to help clinicians with a proper diagnosis.

## Case presentation

**Figure 1:**
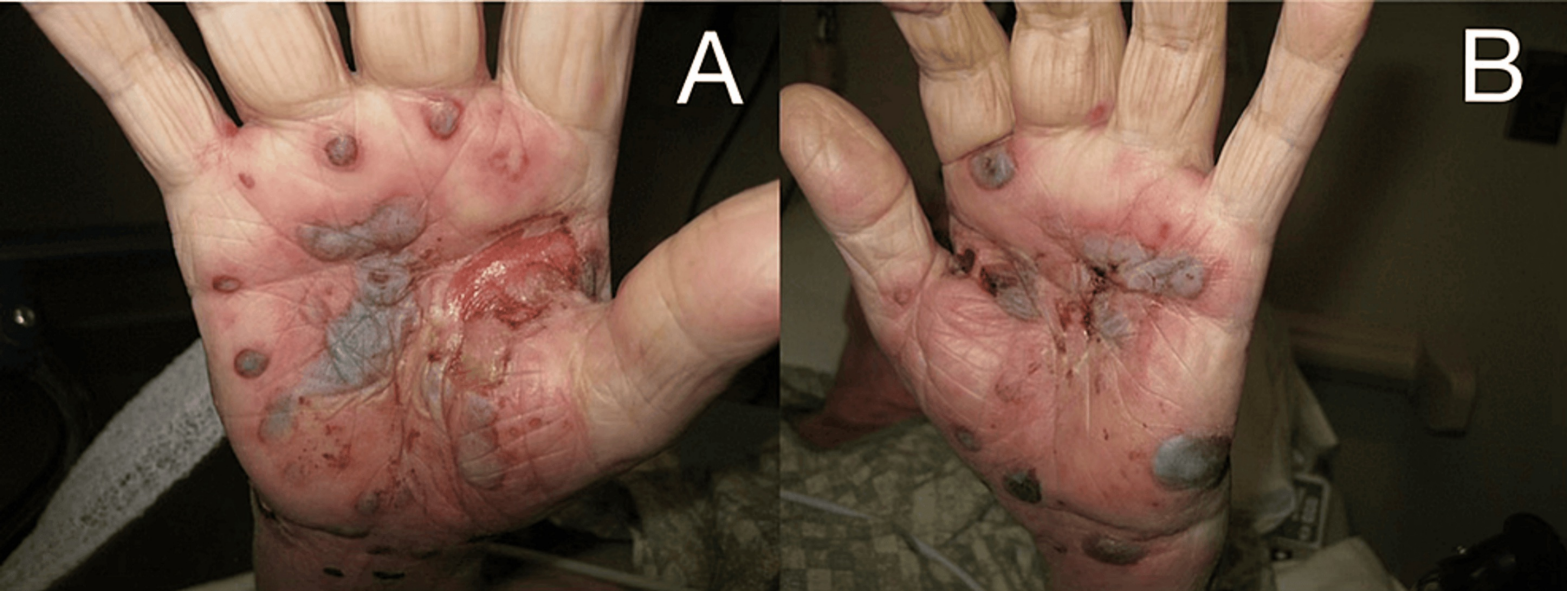
Initial DBP presenting as erythematous, purpuric, hemorrhagic bullous lesions on right (A) and left (B) hands DBP - dyshidrosiform bullous pemphigoid

A 89-year-old male nursing home resident with a history of Alzheimer's disease for over a decade presented with hemorrhagic blisters on his right (Figure 1A) and left (Figure 1B) palms that started 11 days prior to presentation. The patient was not on any medications nor did the patient have any other co-morbidities.

**Figure 2:**
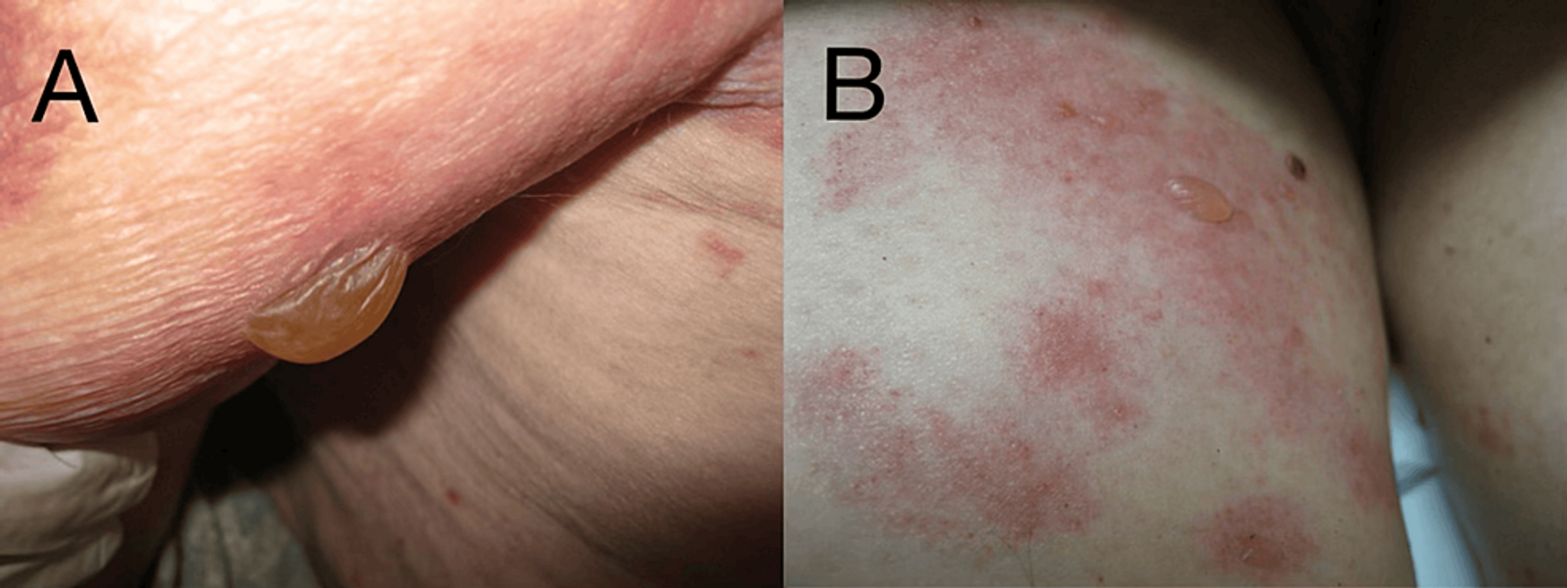
Pruritic erythematous lesions of DBP developed on the arms (A) and thighs (B) at the time of clinical presentation DBP - dyshidrosiform bullous pemphigoid

The patient's family revealed that he had been otherwise healthy, with no recent changes in medication or anticoagulant use. The patient had recently been discharged from a hospital stay and was prescribed augmentin after a wound culture was performed at a different facility. After discharge from his previous facility, blisters on his hands worsened, and he developed pruritic, erythematous lesions on his chest, upper back, lower abdomen, arms (Figure 2A), and bilateral thighs (Figure 2B), prompting representation to the emergency room and admission to the hospital.

**Figure 3:**
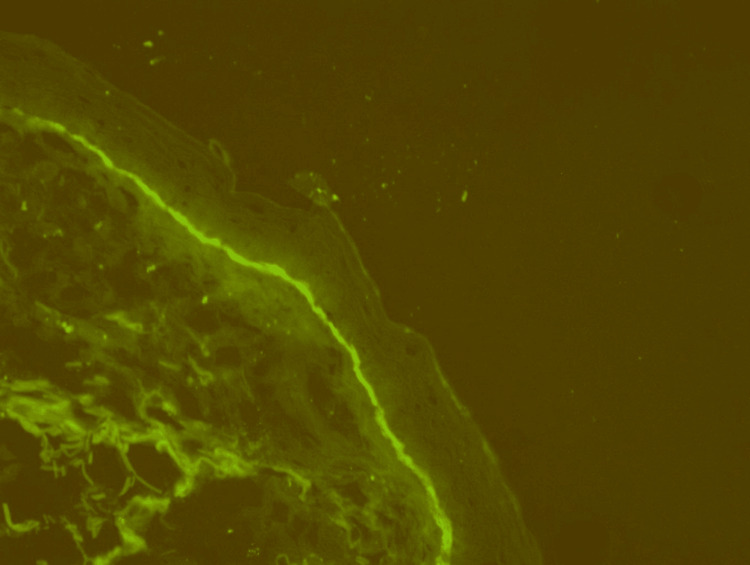
DIF of DBP showing granular IgG and C3 deposits in the basement membrane zone (40x) DIF - direct Immunofluorescence; DBP - dyshidrosiform bullous pemphigoid

To confirm the diagnosis of DBP, direct immunofluorescence (DIF) was performed on the patient. DIF is a commonly used technique that helps identify the presence of autoantibodies and complements within biopsy specimens. In this case, DIF revealed continuous linear deposits of C3 and IgG in the basement membrane zone and underscored the presence of a subepidermal split (Figure 3), which are characteristic findings in DBP [5].

**Figure 4:**
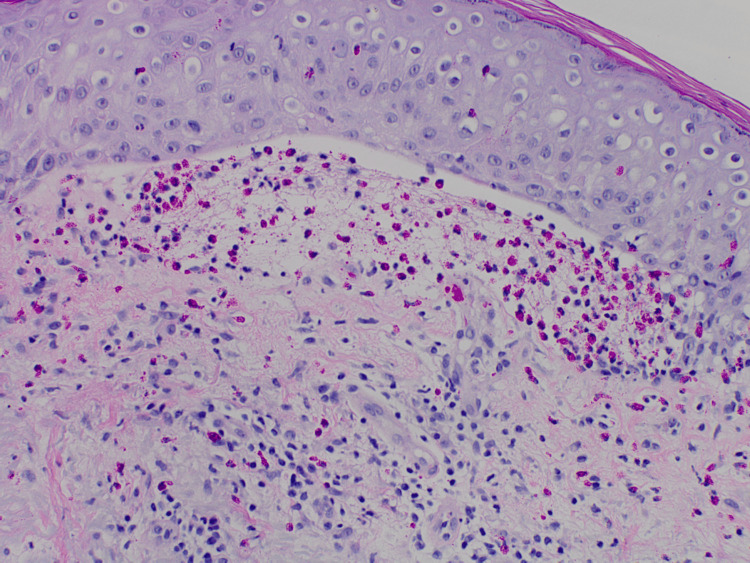
H&E (200x) stain of DBP with characteristic inflammatory eosinophil-rich perivascular and interstitial infiltrates in the papillary dermis DBP - dyshidrosiform bullous pemphigoid; H&E - Hematoxylin and Eosin

Haematoxylin and Eosin (H&E) staining showed focal subepidermal blister formation with numerous eosinophils noted within the dermis and blister cavity (Figure 4).

The patient was started on a prednisone taper while admitted, beginning at 40 mg. He was discharged, and on follow-up two months later, lesions to the trunk and extremities had resolved; however, lesions to the hands were persistent. He was continued on prednisone 30 mg for two weeks and then continued a slow taper of prednisone, while clobetasol 0.05% ointment was prescribed twice daily to the hands. The patient was then seen two months after the initial follow-up, at which time lesions of the hands were recalcitrant, prednisone and clobetasol were continued, and minocycline 100 mg twice daily was added to the treatment regimen. Unfortunately, the patient was unable to follow up before full clearance of the hand lesions.

## Discussion

The treatment for DBP typically involves varying levels of immunosuppression. Topical and systemic corticosteroids, tetracycline antibiotics, dapsone, monoclonal antibodies such as rituximab, dupilumab, and other immunosuppressants such as azathioprine and mycophenolate mofetil may all be used in treatment regimens [1,2,4]. The most widely utilized treatment regimen was systemic corticosteroids (73%), followed by dapsone (35%), oral antibiotics like doxycycline (24%), and immunosuppressants (19%) [6]. Approximately a quarter (24%) of patients had lesions limited to their palms and soles [6].

Topical corticosteroids are frequently the first line of treatment for DB [6]. Their efficacy, however, is limited, and systemic absorption can cause substantial side effects such as hypothalamic-pituitary-adrenal (HPA) axis suppression, iatrogenic Cushing's syndrome, and growth retardation in children [7]. Many individuals require systemic medication to achieve appropriate illness control [7]. According to the literature, a large minority of individuals with DBP do not react to topical corticosteroids and require extra systemic immunosuppression [6].

Dupilumab, a recombinant human IgG4 monoclonal antibody that targets IL-4Rα, has shown promise in treating resistant cases of bullous pemphigoid, including DBP [8]. Dupilumab suppresses Th2-related cytokines, which are increased in BP patients and has been shown to reduce disease activity and steroid reliance [8,9]. An analysis of 30 individuals treated with dupilumab found excellent results, indicating that it is a feasible choice for patients who do not react to traditional therapy [8]. Rituximab, an anti-CD20 monoclonal antibody, has proved effective in treating refractory bullous pemphigoid [9]. It targets B-cells, which play an important role in the development of BP. Rituximab has been demonstrated to be useful in patients who do not react to conventional therapies, such as systemic corticosteroids and immunosuppressants [9]. Omalizumab, a monoclonal antibody targeting IgE, is also being explored as a potential treatment option for DBP [10]. Given the resistant nature of the case mentioned, rituximab or omalizumab may have been evaluated as a therapy option to improve outcomes and quality of life.

In the present case, the patient did not respond adequately to a combination of prednisone, clobetasol ointment, and minocycline for several months. This illustrates the difficulties in controlling DBP with traditional medications and emphasizes the need for more focused therapy. Advances in antibody-based medicines, such as dupilumab and rituximab, have demonstrated promise for improving outcomes for individuals with refractory DBP.

## Conclusions

With limited number of cases on this topic reported in the current literature, the case report provides an overview of the diagnosis and management of DBP in an elderly patient, emphasizing the diagnostic challenges in differentiating DBP from other conditions it may present, such as dyshidrotic eczema, using images to help with diagnosis: cutaneous presentations, DIF, and H&E. The patient's lesions remained after receiving systemic corticosteroids, topical clobetasol, and minocycline as initial treatment, highlighting the need for more focused treatments. For refractory cases, dupilumab and rituximab have demonstrated promise in lowering disease activity and steroid reliance. Since there are limited cases in the current literature, this case seeks to enhance DBP treatment results and diagnostic accuracy by raising physician awareness, which will ultimately lead to better patient care.
